# Sexual behaviors, cannabis, alcohol and monkeypox infection

**DOI:** 10.3389/fpubh.2022.1054488

**Published:** 2023-01-17

**Authors:** Alexandre Vallée

**Affiliations:** Department of Epidemiology-Data-Biostatistics, Delegation of Clinical Research and Innovation (DRCI), Foch Hospital, Suresnes, France

**Keywords:** sexual behavior, cannabis, alcohol, monkeypox, MSM, outbreak, chemsex, epidemic

## Abstract

The emergence of the monkeypox virus (MPXV) outbreak in 2022 is a worldwide health issue. The rapid increase of monkeypox cases caused the WHO to designate the escalating global monkeypox outbreak a Public Health Emergency of International Concern on July 23, 2022. The WHO has called on the group currently most affected by the virus, men who have sex with men (MSM), to limit their sexual partners. The diminution in number of sexual partners not only decreases the proportion of infected MSM but could also increases the number of days needed to reach a given infection level among the general population. Several behavioral factors could be associated with high levels of different sexual partners, such as cannabis use and alcohol consumption. Firstly, this review focuses on the association between cannabis and alcohol consumption and the number of sexual partners, and their possible impact on the current MPXV outbreak by impairing the immune responses. Secondly, this review investigated in the UK Biobank cohort the relationship between alcohol and cannabis use and the number of sexual partners. Among the 115,604 participants, 1.8% declared to be MSM, 1.9% to be WSW (women having sex with women), 43.3% men heterosexuals and 53.0% women heterosexuals. MSM and WSW showed higher lifetime sexual partners (*N* = 17.4 (SD:17.52) and *N* = 13.65 (SD: 13.21), respectively) compared to heterosexual men (*N* = 6.89 (SD: 9.47) and women (*N* = 5.19 (SD:6.56), *p* < 0.001. After adjustment for age, body mass index, lifetime sexual activity, educational and income levels, tobacco and cardiovascular diseases, cannabis use and alcohol consumption remained significantly associated with increase in the number of different sexual partners in all four subgroups. Thus, cannabis use and alcohol consumption may have two detrimental effects on the MPXV outbreak: by participating in the increase of the number of sexual partners which are mainly responsible for the augmentation of the number of new MPXV infected cases and by impairing the immune response to a viral infection. Health and safety policies should address the factors and practices, including chemsex, leading to an increase in risk of sexual behaviors responsible for MPXV dissemination in the worldwide population.

## Introduction

The emergence of the monkeypox virus (MPXV) outbreak in 2022 is a worldwide health issue (1). To date, more than 58,000 laboratory-confirmed cases and 18 deaths have been observed by the World Health Organization (WHO) from 103 territories in all six WHO Regions (2). The rapid increase in monkeypox infected cases caused the WHO to designate the escalating global monkeypox outbreak a Public Health Emergency of International Concern on July 23, 2022. Like previous infectious diseases (3), Monkeypox is a contagious disease which requires physical or sexual contact with people infected with the virus (4). Although the risk to the overall population is considered to be rather low, the WHO has responded by making this pandemic as a high priority to avoid further outbreaks (5). In a recent report, the WHO has called on the group currently most affected by the virus, men who have sex with men (MSM), to limit their sexual partners. Recent analyses showed numerous risk determinants, such as being young men, having sex with other men (MSM), having risk behavioral attitudes and activities, including condomless sex, PrEP (pre-exposure prophylaxis) and HIV positivity (6, 7). Indeed, the transmission of MPXV has been correlated with close relationships, especially sexual contact, between men (4, 8) but also within heterosexual populations (9). Studies have suggested that gay, bisexual, and other MSM have taken steps to protect themselves and their sexual partners against MPXV, such as reducing their number of sexual relationships (10, 11). Thus, the decrease in the number of sexual partners not only decreases the percentage of infected MSM but could also increase the number of days needed to reach a given infection level among the overall population. This could allow more time for vaccination campaigns to reach targeted people (12). The decrease in sexual partners could significantly decrease the MPXV transmission rate (11) and slow down the trend toward a pandemic (13). Several behavioral comportments could be associated with high levels of different sexual partners, such as cannabis use (14) and alcohol consumption (15). Thus, this review focuses on the association between cannabis and alcohol consumption on the number of sexual partners, sexual behaviors, and their possible impact on the current MPXV outbreak.

### Monkeypox virus outbreak

**Figure 1:**
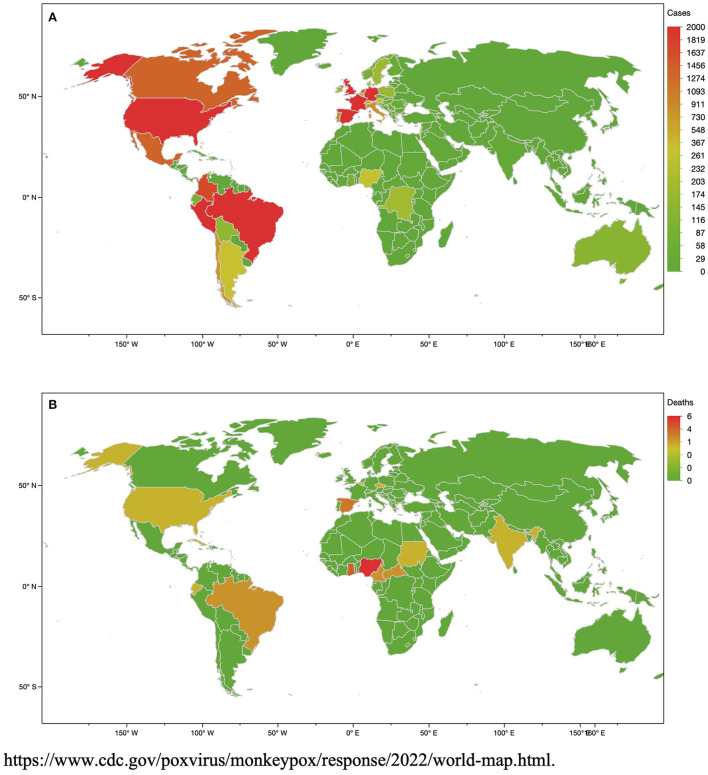
Number of monkeypox virus (MPXV) cases **(A)** and deaths **(B)** at the date of 23th September, 2022.

Monkeypox (MPX) is a zoonotic viral disease which originates from the monkeypox virus (MPXV). This disease has been known for over 50 years but was limited to a restricted number of endemic territories localized in Central and West Africa. Nevertheless, since the years 2000, sporadic reports of imported cases have been observed in North America, Europe, and the Middle East. To date, a worldwide epidemic has shown major issues as the disease is quickly spreading, mainly in young MSM, showing a classic vesicular-pustular rash along with other clinical symptoms (16). More than 65,000 laboratory-confirmed cases and 26 deaths have been observed by the World Health Organization (WHO) from all the territories in the WHO Regions (Figure 1) (2)^1^. The skin is the major source of infection and contamination (17). Although respiratory droplets are thought to diffuse the infection from person to person, the US CDC (Centers for Disease Control and Prevention) declared that the transmission requires a long face-to-face relationship due to the inability of the droplets to cover a long distance. Whereas MPXV remains not only sexually transmitted by vaginal or sperm secretions, health authorities have declared that the current epidemic is due to human-to-human sexual intercourse (4, 18). MPXV is observed in seminal fluid, genital and rectal lesions, and feces and saliva from confirmed infected people in several countries (4, 19). Thus, human-to-human propagation needs contact with lesions, respiratory droplets, or bodily fluids. MPXV infection is characterized by first, a fever associated with headaches, body aches and asthenia. Two days after the fever, a blistering rash begins with formation of scab and scarring. The vesicles are mainly localized on the hands, the palms and the face, and the soles of the feet but can also be present around the mouth and the genital area. The incubation of the MPX ranges from 5 to 21 days. The MPX usually heals spontaneously after 2 to 3 weeks (20).

### Monkeypox infection and its complications

A recent French observational study found that complications affected more than a third of the patients (21). The main frequent complications were anal pain, and secondary bacterial skin infections, including cellulitis. The common neurologic symptoms were prodromal frontal headache occurring in the majority of patients (22) in association with asthenia and myalgias. Conjunctivitis can occur with corneal lesions and vision loss (23). Encephalitis has been observed in rare cases. Other complications can include pneumonitis, keratitis and secondary bacterial infections (23). Few patients were hospitalized but can represent up to 6% of them (21). From the beginning of the worldwide 2022 outbreak, the WHO reported 20 confirmed deaths for 64,290 cases in September, 2022 (24), and only six deaths have been reported in the United States for more than 25,000 cases (25). These data are consistent with the mortality rates of past MPXV outbreaks in Africa ranging from 1% in Nigeria to 10% in the Congo Basin (23). Most of the deaths have occurred in young people and people living with HIV (26).

### Cannabis use

Marijuana (cannabis) is the main worldwide consumed illicit drug, with over 188 million users, or around 2.5% of the population of 15–64 years (27). Cannabis use is correlated with poor economic, social, psychosocial and health levels. The psychosocial repercussions of cannabis use have been well reviewed (27), with drop out of school, antisocial behaviors and poor school performance. The health repercussions of cannabis use implicate several physiological and biochemical processes including immune, cardiovascular, hepatic, renal, endocrine and general health issues (28). Furthermore, around 38 million people are living with HIV, 170 million with hepatitis C virus, and 10 to 20 million with T-lymphotropic virus type 1. Most of them are cannabis users (29).

### Alcohol consumption

More than 100 million people worldwide are observed to have inappropriate alcohol consumption (30). Globally, 3 million deaths per years are the consequences of alcohol disorder. 5.3% of all causes of death are represented by alcohol disorder. Overall, 5.1% of the global burden of disease and injury is attributable to alcohol. Chronic alcohol consumption was associated with progression of community infections and complications in COVID-19 (31).

### Cannabis, alcohol, and sexual behaviors

Several studies have shown that the relationship between alcohol and sexual behaviors increases the probability of HIV transmission and the absence of use of condoms in anal sexual practice (32–34). Among MSM, the consumption of alcohol is a major determinant of viral infection, such as HIV (33, 35). Nevertheless, the information regarding the effect of cannabis is limited (36). Around 40% of MSM reported to be heavy cannabis users, in comparison to seven percent of the overall population (37). Furthermore, in young MSM, the cannabis is more consumed than alcohol (daily 23 vs. 2%) (38). Recent investigations have suggested that MSM consume cannabis before having sex as frequently as alcohol consumption (63.5 vs. 61.5%) (39). Evidence also suggested that cannabis could increase condomless sex in heterosexual people (40–43).

The delta-9-tetrahydrocannabinol (THC) which composed cannabis could generate pharmacological actions on sexual-risk decision-making as could the consumption of alcohol (44). THC can lead to euphoric mood, impulsivity, risk-taking and aphrodisiac effects and can diminish the capacity of initiating responses (45, 46). Some studies have shown that, among MSM, cannabis and alcohol consumption may affect cognitive functions (47, 48). This risk is higher than the drug alone, with higher behavioral and social impacts and distress (34). Similar results have been observed among heterosexual young people, with higher risk of condomless sex (49, 50). In contrast, other studies have shown inconclusive results (36, 38, 51). The decision to use condoms or not may be impacted by other HIV prevention strategies, including pre-exposure prophylaxis (PrEP) and treatment-as-prevention (TasP), which could diminish the risk of sexually-based HIV infection, even in case of condomless sex (52, 53). The relationship between alcohol and sexual risk taking has been widely shown (54, 55). Alcohol consumption is correlated with disinhibition of behavioral sex comportments, leading to highly risk of exposure to sexually transmitted infections for individuals under its influence (56, 57). Alcohol consumption, especially in individuals who drink to intoxication levels, is correlated with higher risk of condomless sex (58, 59). Face to the MPXV outbreak, the WHO has called MSM to limit their sexual partners. In this review, we point the fact that several studies have shown that cannabis use was correlated with higher numbers of sexual partners for both genders (60, 61). Sexually transmitted diseases remain a major health issue, and new cases of infection are mainly attributable to the sexual relationships, among both MSM, women having sex with women (WSW) and heterosexuals (58). Thus, it is essential to investigate the role of both alcohol consumption and cannabis use with the number of sexual relationships in these different subgroups of populations.

### Cannabis use, alcohol consumption and number of sexual partners, data from the UK Biobank

The objective of this part of the work was to confirm the review information by original results of the association between cannabis use and alcohol consumption and the number of sexual partners among MSM, WSW and heterosexual men and women of the UK Biobank.

## Materials and methods

### UK biobank population

The UK Biobank is a prospective cohort for the investigation, prevention, diagnosis, and treatment of chronic diseases, such as CV diseases in adults. 502,478 Britons aged of 40–70 years across 22 UK cities from the UK National Health Service Register were included between 2006 and 2010. The cohort was phenotyped and genotyped, with participants who responded to a questionnaire; a computer-assisted interview; physical and functional measures; and blood, urine, and saliva samples (62–64). Data included socio-economic status, lifestyle behaviors, a mental health battery, clinical diagnoses and therapies, genetics, imaging and physiologic biomarkers from blood and urine samples. The cohort protocol can be found in the literature (65).

### Lifetime number of sexual partners

Sexual partners were reported by questionnaire. Participants were asked: “About how many sexual partners have you had in your lifetime?”. Lifetime sexual activity was defined as the difference between age at inclusion and the age of first sexual intercourse. Men having sex with men (MSM) and women having sex with women (WSW) were defined as participants who declared having same sex intercourse.

### Cannabis use

Cannabis use was reported by questionnaire. Participants were asked about their life-time cannabis use: “Have you taken cannabis (marijuana, grass, hash, ganja, blow, draw, skunk, weed, spliff, dope), even if it was a long time ago?”. Those who responded “no” were classified as controls (i.e., never users) and those endorsing “yes” options were classified as cannabis users. We separated these users into three groups: those reporting initial cannabis use (“yes, 1–2 times”, or “yes, 3–10 times”: low users) and continued cannabis use (“yes, 11–100 times”: moderate users; and “yes, more than 100 times”: high users).

### Alcohol consumption

Although the alcohol questionnaire has not been formally validated, several studies have shown expected associations with alcohol (66, 67). Alcohol level consumption was defined as reported in the questionnaire: high level (“daily or almost daily” or “three or four times a week”), moderate level (“once or twice a week,” or “one to three times a month”), and low level (“special occasions only” or “never”). Then participants self-reported the number of alcohol units (10 ml of pure ethanol) consumed, in “units per week” or “units per month” (for less frequent drinkers), across numerous beverage categories (red wine, white wine/champagne, beer/cider, spirits, fortified wine, and “other”). The UK Biobank assessment defined units of alcohol as: a pint or can of beer/lager/cider = two units; a 25 ml single shot of spirits = one unit; and a standard glass of wine (175 ml) = two units. The number of weekly units by summing the weekly units consumed in all categories was computed. When reported monthly, the intake was converted to units per week by dividing by 4.3. The number of weekly units was divided by 7 to determine units per day (68).

### Covariates

Current tobacco smokers were defined as participants who responded “yes, on most or all days” or “yes, only occasionally” to the question “do you smoke tobacco now.” Smoking pack-years are calculated as the average number of packs smoked per day multiplied by the total number of years smoking during lifetime. Body mass index was calculated as weight (in kg) divided by height^2^ (meters), and categorized as high (BMI > 30 kg/m^2^), moderate (BMI between 25 and 30 kg/m^2^) and low (< 25 kg/m^2^). Cardiovascular (CV) diseases were defined by heart attack, angina and stroke, as diagnosed by a doctor and reported in the questionnaires (69). Education level was defined in three categories, high (college or university degree), intermediate (A/AS levels or equivalent, O levels/GCSEs or equivalent), and low (none of the aforementioned) (70). Income level was defined as, high (“>£52,000 per year”), moderate (between £18,000 and £51,999 per year), and low (“ < £18,000 per year”) (71).

### Ethical considerations

All participants provided electronic informed consent and UK Biobank received ethical approval from the North-West Multi-center Research Ethics Committee (MREC) covering the whole of the United Kingdom. The study was conducted in accordance with the guidelines of the Declaration of Helsinki and approved by the North-West–Haydock Research Ethics Committee (protocol code: 21/NW/0157, date of approval: 21 June 2021). For details: https://www.ukbiobank.ac.uk/learn-more-about-uk-biobank/about-us/ethics.

### Study population

Inclusion criteria were participants who responded to the cannabis use questionnaire, to the questionnaire of number of sexual partners, and to alcohol consumption per day. Exclusion criteria were missing data for all covariates (like age, lifetime sexual activity, gender, income level, educational level, smoking pack years), 115,604 participants were included in the study.

### Statistical analysis

Characteristics of the study population were described as the means with standard deviation (SD) for continuous variables. Categorical variables were described as numbers and proportions. To compare characteristics among the quartiles, we used the one-way ANOVA test for continuous variables and the chi-square test for categorical variables. Comparisons between two groups were performed using Student's test for continuous variables. Pearson's χ^2^ test was performed for categorical variables. Multivariate linear regression models were performed for the relationship between cannabis use and alcohol consumption, with adjustment for age, BMI, lifetime sexual activity, education, income, smoking pack years and CV diseases. Statistics were performed using SAS software (version 9.4; SAS Institute, Carry, NC). A *p* < 0.05 was considered statistically significant.

## Results

**Figure 2:**
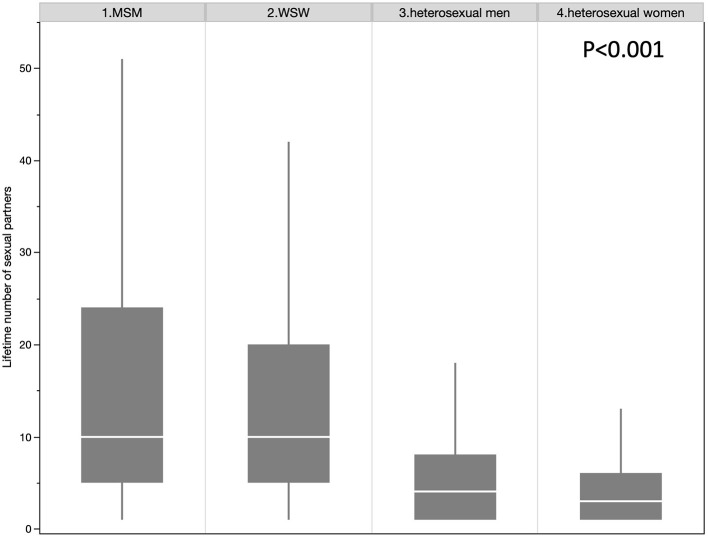
Lifetime number of sexual partners between the four groups.

**Figure 3:**
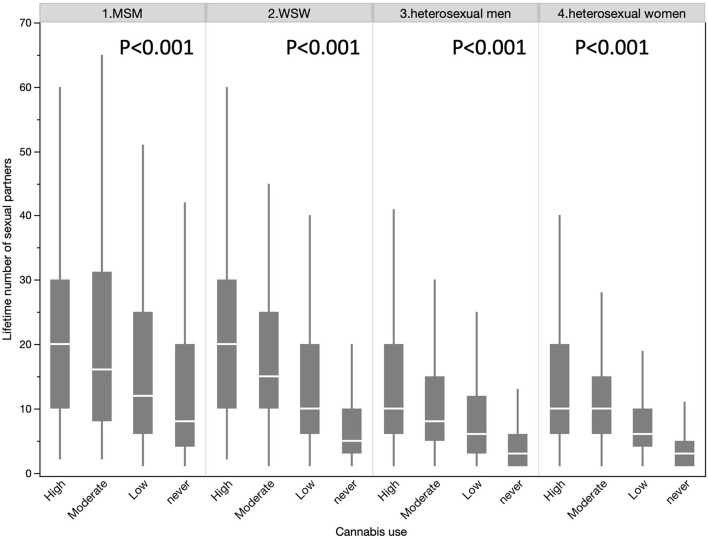
Lifetime number of sexual partners in all the subgroups according to cannabis use.

**Figure 4:**
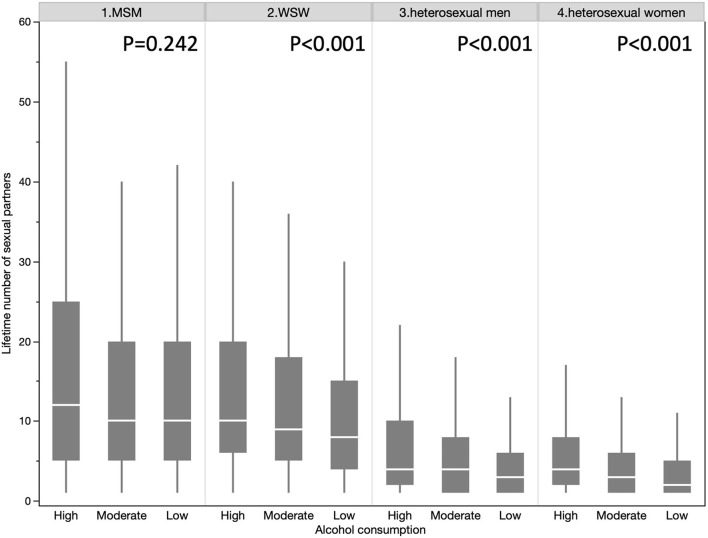
Lifetime number of sexual partners in each groups according to alcohol consumption level.

Among the 115,604 participants, 2,059 (1.78%) declared to be MSM, 2,243 (1.94%) to be WSW, 50,007 (43.26%) to be heterosexual men and 61,295 (53.02%) to be heterosexual women (Table 1). The gay population (both MSM and WSW) showed a significantly higher number of different sexual partners than the heterosexual population (15.4 vs. 6.0, *p* < 0.001). The gay population has a higher proportion of high cannabis users than the heterosexual population (10.58 vs. 2.356%, *p* < 0.001) and higher levels of alcohol consumption (27.96% vs 23.77%, p < 0.001). The same results were observed by stratifying by gender (Figure 2). The number of different sexual partners increases with cannabis use in all the groups, *p* < 0.001 (Figure 3). However, as the number of different sexual partners increases with alcohol consumption in heterosexual women (*p* < 0.001) and men (*p* < 0.001), and among WSW (*p* < 0.001), but this was not the case among MSM (*p* = 0.242) (Figure 4).

**Table 1:** Characteristics of the study population.

	**MSM**		**WSW**		**Heterosexual men**		**Heterosexual women**		***P* value**
	***N*** = **2,059**		***N*** = **2,243**		***N*** = **50,007**		***N*** = **61,295**		
**Age (years)**	53.36	8.03	51.83	7.31	56.45	7.77	55.07	7.62	< 0.001
**Number of sexual partners**	17.38	17.52	13.65	13.21	6.89	9.47	5.19	6.56	< 0.001
**Age first intercourse**	19.50	6.13	17.99	4.45	19.75	4.88	19.04	4.10	< 0.001
**Lifetime sexual activity**	33.85	9.82	33.84	7.86	36.69	8.66	36.02	7.94	< 0.001
**Average alcohol daily consumption (units/days)**	3.03	3.06	2.12	2.32	2.943	2.73	1.672	1.85	< 0.001
**Cannabis use**									< 0.001
High	227	11.02%	228	10.16%	1,746	3.49%	873	1.42%	
Moderate	210	10.20%	350	15.60%	2,659	5.32%	2,159	3.52%	
Low	597	28.99%	733	32.68%	8,169	16.34%	8,500	13.87%	
Never	1,025	49.78%	932	41.55%	37,433	74.86%	49,763	81.19%	
**Alcohol level**									< 0.001
High	639	31.05%	563	25.12%	14,580	29.16%	11,875	19.38%	
Moderate	1,160	56.37%	1,338	59.71%	30,897	61.80%	38,839	63.38%	
Low	259	12.59%	340	15.17%	4,522	9.04%	10,570	17.25%	
**Educational level**									< 0.001
High	1,228	59.64%	1,564	69.73%	25,535	51.06%	29,682	48.42%	
Moderate	575	27.93%	512	22.83%	15,314	30.62%	23,452	38.26%	
Low	256	12.43%	167	7.45%	9,158	18.31%	8,161	13.31%	
**Income level**									< 0.001
High	784	38.08%	882	39.32%	19,524	39.04%	19,999	32.63%	
Moderate	951	46.19%	1,055	47.04%	25,451	50.89%	32,346	52.77%	
Low	324	15.74%	306	13.64%	5,032	10.06%	8,950	14.60%	
**BMI (kg/m** ^**2**^ **)**	27.13	4.52	26.60	5.26	27.26	3.95	26.31	4.88	< 0.001
**BMI level**									< 0.001
High	427	20.78%	474	21.21%	10,289	20.62%	11,269	18.42%	
Moderate	925	45.01%	746	33.38%	24,668	49.44%	21,395	34.97%	
Low	703	34.21%	1,015	45.41%	14,938	29.94%	28,518	46.61%	
**Current tobacco**	204	9.91%	206	9.18%	2,356	4.71%	2,479	4.04%	< 0.001
**Smoking pack years (pack-years)**	9.87	17.31	6.88	12.07	6.76	14.14	4.07	9.81	< 0.001
**CV diseases**	98	4.77%	27	1.21%	2,764	5.53%	1,070	1.75%	< 0.001

Continuous variable as mean and standard deviation, categorical variable as number and percentage.

After adjustment for age, BMI, lifetime sexual activity, education, income, smoking pack years and CV diseases, the number of different sexual partners was significantly higher in high cannabis users compared to never users in all the groups (MSM, *p* = 0.031; WSW, *p* < 0.001; heterosexual women, *p* < 0.001 and heterosexual men, *p* < 0.001). The same results were observed for average daily alcohol consumption (MSM, *p* = 0.004; WSW, *p* < 0.001; heterosexual women, *p* = 0.002 and heterosexual men, *p* < 0.001) (Table 2). No interactions were observed between cannabis use and alcohol consumption in MSM (*p* = 0.674) and in WSW (*p* = 0.362) but significant interactions were observed in heterosexual women (*p* = 0.047) and in heterosexual men (*p* < 0.001).

**Table 2:** Multivariate linear regression models for cannabis use and alcohol consumption with lifetime number of sexual partners, with adjustment for age, BMI, lifetime sexual activity, education, income, smoking pack years and CV diseases.

**MSM**	**Beta (95%CI)**	***P* value**
Cannabis		
*High*	2.03 (0.18; 3.87)	0.031
*Moderate*	2.70 (0.84; 4.57)	0.005
*Low*	−0.47 (−1.81; 0.85)	0.481
*Never*	Ref.	
Alcohol consumption	0.37 (0.12; 0.61)	0.004
**WSW**	**Beta (95%CI)**	***P*** **value**
Cannabis		
*High*	6.01 (4.72; 7.29)	< 0.001
*Moderate*	2.27 (1.19; 3.34)	< 0.001
*Low*	−2.14 (−2.99; −1.28)	< 0.001
*Never*	Ref.	
Alcohol consumption	0.26 (0.04; 0.48)	0.020
**Heterosexual men**	**Beta (95%CI)**	***P*** **value**
Cannabis		
*High*	3.99 (3.66; 4.31)	< 0.001
*Moderate*	1.19 (0.91; 1.46)	< 0.001
*Low*	−0.89 (−1.09; −0.69)	< 0.001
*Never*	Ref.	
Alcohol consumption	0.32 (0.29; 0.35)	< 0.001
**Heterosexual women**	**Beta (95%CI)**	***P*** **value**
Cannabis		
*High*	4.09 (3.79; 4.39)	< 0.001
*Moderate*	2.13 (1.92; 2.35)	< 0.001
*Low*	−1.11 (−1.26; −0.97)	< 0.001
*Never*	Ref.	
Alcohol consumption	0.27 (0.25; 0.30)	< 0.001

### Limitations

The cross-sectional observational design limits the relationship of causality. Reverse causation cannot be ruled out. The number of sexual partners was self-reported and could be considered as a major bias. Moreover, no historical indication of sexually transmitted infections has been reported in the UK Biobank cohort and could not allow us to investigate the relationship between number of sexual partners and sexually transmitted infection prevalence.

### Cannabis and alcohol with immune and viral systems

It is well-known that illicit or licit substances could affect several components of the immune system, by dysregulating the function of distinct immune response cells. Numerous studies have shown that some drugs could influence lymphocytes as well as dendritic cells and macrophages. Cannabis use, as well as alcohol consumption, has been reported to damage immune responsiveness. The recreational use of such drugs has been well described to affect resistance to microorganisms and alter susceptibility to infectious diseases (72). The consumption of cannabis and alcohol simultaneously is known to dysregulate the inflammatory responses through toll-like receptors (TLRs) (73).

Cannabidiol (CBD), one of the compound of cannabis, can damage the functional roles of the immune system (74). Cannabis acts as an immune modulator, damaging T-cells, B-cells, monocytes, and microglia, leading to reduction of the production of proinflammatory cytokines and an increase in the activity of anti-inflammatory cytokines. The role of cannabis immunity has been well discussed (75, 76). Cannabis use could predispose people to pulmonary infection, in patients showing a decrease in immune defenses by HIV infection chemotherapy (77). These people show that cannabis generates a concentration-dependent diminution in the proliferation of T cells and in the production of IFN-γ through CB2 receptor-dependent signaling. At the level of gene expression, cannabis stimulates Th1 cytokines (IFN-γ/IL-2) expression and decreasesTh2 cytokine (IL-4/IL-5) expression. Thus, both consumption of cannabis and alcohol significantly stimulates the production of IL-6 cytokine and toll-like receptors, TLR5, TLR7 and TLR9s. This suggests cannabis-related action on pulmonary innate immunity promoting airway inflammation (73). People living with viral infections can consume several illegal drugs, including cannabis. With regard to its negative actions, there are investigations which support the information that cannabis is detrimental for viral infections. Recent studies have shown that cannabis could control the immune system by modulating specific receptors on immune cells and decreasing immunity (78–83). Nevertheless, several investigations have shown that cannabis could have some positive actions (27). Thus, cannabis could affect the immune system (74, 75), could stimulate inflammatory cytokine production and CRP levels (84), and could decrease resistance to viral infections (85, 86). However, the literature remains inconsistent for the alteration of T cell lymphocytes, B cell lymphocytes, macrophages or immunoglobulin (27). Cannabis may reduce inflammation in HIV infection (87), and impairment of neurocognition of people living with HIV (88).

Similar results were observed for alcohol consumption. Alcohol may exert a dose-dependent interaction on the host response to viral infection (89). High alcohol consumption increases viral and bacterial infections (90), severity of infections (91), leads to viral infection expression, including HIV (92) and hepatitis C (93). However, moderate alcohol consumption was correlated with enhancement of immune response to infection and vaccination (94–96). The negative actions of high alcohol consumption on the immune system have been widely shown (97). Alcohol consumption can affect cell-mediated immunity and can be associated with high levels of post-operative infections (98). Ethanol diminishes cytokine production, affects macrophage responses to cytokines and LPS and stimulates the intracellular survival rate of Mycobacteria, Legionella, Salmonella or Listeria (99).

### Cannabis and orthopoxvirus infection

Few investigations have reported cannabis use and orthopoxvirus infection, none with the MPXV infection (100, 101). A team from Austria reported in 2016 that a young patient infected by an unusually severe cowpox virus (CPXV) with generalized rash and fever (102), showed abused substances associated with severe symptoms and diminution of the overall anti-CPXV antibody titer (100). They also showed that a single dose of cannabinoids increased the severity of CPXV infection in animals (101). Thus, a monodose of cannabis resin or THC before infection was associated with the reduction of the anti-CPXV anti-body formation in mice. The patient viewed by this team, developed a severe and generalized CPXV infection but without antibody response (100). The production of antibodies may have a major and protective role in CPXV infections (103). However, data are not consistent for the role of cannabis in antibody formation. A recent investigation showed a correlation between the diminution of immunoglobulin G and M rates and cannabis use (104) while other studies did not observe modifications in B-cell numbers rather than augmented IgE levels (105).

In a report of CPXV infection, the patient presented levels of lymphocytes within a normal range and serum analysis showed normal levels of immunoglobulin IgM but augmented serum IgG and IgE levels (100). These observations could be mainly attributed to a cannabinoid drug action showing an augmentation of the levels of IgE (99). Cannabis use can lead to immune-suppression leading to an enhancement of susceptibility to infection (100).

An association between cannabis use and herpes virus infections has been shown to reduce the phagocytic ability of alveolar macrophages, the NK-cell activity, interferon-gamma, and interleukin IL-12 levels (106). This observation of an increase of intracellular agent survival rate, and then of a decrease in survival rate of THC-treated mice with Legionella pneumophila infection (106).

Cannabis can influence the humoral immune response (107) and can control allergic immune responses (108) and neuro-inflammatory disorders (109). Even if no data clearly showed a negative role of cannabis in Orthopoxvirus, and its detrimental actions in viral infections, the significant reduction of Ig levels against vaccines and the reduction of complement protein in specific populations, like students, can highlight the dimension of the health problem (104).

No data linked alcohol consumption and Orthopoxvirus infection.

### Chemsex, alcohol, cannabis and monkeypox infection

The incidence of STIs (sexually transmitted infections) has globally increased and continues to bear a disproportionate disease burden (110). Chemsex is sexualized drugs use and could be characterized as the intention to use illicit substances and/or drugs to enhance pleasure during sex. These substances include gamma-hydroxybutyrate (GHB), crystal meth, mephedrone, ecstasy, or cocaine (111). Chemsex is associated with a higher risk of reduction in the use of condoms (112, 113), increased risk of multiple sexual partners (114, 115) and higher risk of STI transmission, including HIV (116, 117). Face to the MPXV outbreak, chemsex practice could be a high-risk sexual practice for virus transmission. Furthermore, recent studies have observed that chemsex is often associated with cannabis use (118) and alcohol consumption (44).

Although investigations were mainly focused on LGBT (gay, lesbian, bisexual and transgender) populations, recent studies have shown that the practice of chemsex could be also frequently observed in both women and men, regardless of their sexual orientation (119, 120). Nevertheless, certain drug combinations should be considered according to the different populations. For example, alcohol and cannabis were mainly used in association with ecstasy among heterosexual sexual relationships (121, 122). To date, very few studies have investigated the role of chemsex in the MPXV outbreak. Wang et al. (123), showed that MSM using chemsex recently were less likely to perceive higher concern for MPXV infection (123). One of the explanations could be that MSM using chemsex tend to underestimate the STI risk in general (124, 125). However, Thornhill et al. (20), reported a prevalence of 20% of chemsex use among 528 infections diagnosed in 16 countries (20). Moreover, in a French cohort of patients infected by MPXV, 42% (90/216) reported having practiced chemsex in the last 3 months, and 40% (106/264) having condomless sex (21).

## Conclusion

The emergence of the monkeypox virus (MPXV) outbreak in 2022 has become a worldwide health issue. Monkeypox is a contagious disease which requires physical or sexual contact with someone infected with the virus. In a recent report, the WHO has called on the group currently most affected by the virus, men who have sex with men (MSM), to limit their sexual partners. In this review, we observed that cannabis use and alcohol consumption are mainly correlated with a high number of sexual partners and at-risk sexual behaviors in both gay and heterosexual populations, which can lead to increase the dissemination of the MPXV and therefore lead to a sharp increase in this outbreak. Cannabis use and alcohol consumption may have two detrimental effects for the MPXV outbreak: by participating in the increase of the number of sexual partners which is mainly responsible for the augmentation of the number of new MPXV infected cases and by impairing immune responses to a viral infection. Health preventing policies should address the factors and practices leading to an increase in risk of sexual behaviors responsible for MPXV dissemination in the worldwide population (126). Health professionals should be aware of all risk behaviors concerning the MPXV outbreak to implement appropriate health policies without stigmatization to prevent discrimination and optimize compliance to these messages.

## Author contributions

AV: conceptualization, methodology, formal analysis, and writing—original draft preparation.

## Footnotes

^1^ https://www.cdc.gov/poxvirus/monkeypox/response/2022/world-map.html
